# Long-Term Risk of Arterial Thrombosis After Intracerebral Hemorrhage: MUCH-Italy

**DOI:** 10.1161/STROKEAHA.123.044626

**Published:** 2024-02-01

**Authors:** Alessandro Pezzini, Licia Iacoviello, Augusto Di Castelnuovo, Simona Costanzo, Barbara Tarantino, Giovanni de Gaetano, Marialuisa Zedde, Simona Marcheselli, Giorgio Silvestrelli, Alfonso Ciccone, Maria Luisa DeLodovici, Lucia Princiotta Cariddi, Maurizio Paciaroni, Cristiano Azzini, Marina Padroni, Massimo Gamba, Mauro Magoni, Massimo Del Sette, Rossana Tassi, Ivo Giuseppe De Franco, Anna Cavallini, Rocco Salvatore Calabrò, Manuel Cappellari, Elisa Giorli, Giacomo Giacalone, Corrado Lodigiani, Mara Zenorini, Francesco Valletta, Rosario Pascarella, Ilaria Grisendi, Federica Assenza, Manuela Napoli, Claudio Moratti, Maurizio Acampa, Mario Grassi

**Affiliations:** 1 Dipartimento di Medicina e Chirurgia, Università degli Studi di Parma, Italy (A.P.).; 2 Programma Stroke Care, Dipartimento di Emergenza-Urgenza, Azienda Ospedaliera Universitaria, Parma, Italy (A.P.).; 3 Dipartimento di Epidemiologia e Prevenzione, IRCCS Neuromed, Pozzilli, Italy (L.I., S.C., G.G.).; 4 Dipartimento di Medicina e Chirurgia (L.I.), Università dell’Insubria, Varese, Italy.; 5 Unità di Neurologia, Ospedale di Circolo (M.L.D.L., L.P.C.), Università dell’Insubria, Varese, Italy.; 6 Mediterranea Cardiocentro, Napoli, Italy (A.D.C.).; 7 Dipartimento di Scienze del Sistema Nervoso e del Comportamento, Unità di Statistica Medica e Genomica, Università di Pavia, Italy (B.T., M. Grassi).; 8 S.C. Neurologia, Stroke Unit (M. Zedde, I.G., F.A.), AUSL-IRCCS di Reggio Emilia, Italy.; 9 SSD Neuroradiologia (R.P., M.N., C.M.), AUSL-IRCCS di Reggio Emilia, Italy.; 10 Neurologia d’Urgenza and Stroke Unit (S.M.), IRCCS Istituto Clinico Humanitas, Rozzano-Milano, Italy.; 11 UOC Centro Trombosi e Malattie Emorragiche (C.L.), IRCCS Istituto Clinico Humanitas, Rozzano-Milano, Italy.; 12 Stroke Unit, Dipartimento di Neuroscienze, ASST Mantova, Italy (G.S., A. Ciccone).; 13 Stroke Unit and Divisione di Medicina Cardiovascolare, Università di Perugia, Italy (M. Paciaroni).; 14 Stroke Unit, Divisione di Neurologia, Dipartimento di Neuroscienze e Riabilitazione, Azienda Ospedaliero-Universitaria di Ferrara, Italy (C.A., M. Padroni).; 15 Stroke Unit, Neurologia Vascolare, Spedali Civili di Brescia, Italy (M. Gamba, M.M.).; 16 U.O. Neurologia, IRCCS Policlinico San Martino, Genova, Italy (M.D.S.).; 17 Stroke Unit, AOU Senese, Siena, Italy (R.T., I.G.D.F., M.A.).; 18 UOC Malattie Cerebrovascolari e Stroke Unit, IRCCS Fondazione Istituto Neurologico Nazionale “C. Mondino,” Pavia, Italy (A. Cavallini).; 19 Istituto di Ricovero e Cura a Carattere Scientifico, Centro Neurolesi Bonino-Pulejo, Messina, Italy (R.S.C.).; 20 Stroke Unit, DAI di Neuroscienze, Azienda Ospedaliera Universitaria Integrata Verona, Italy (M.C., M. Zenorini, F.V.).; 21 U.O. Neurologia, Ospedale S. Andrea, La Spezia, Italy (E.G.).; 22 Stroke Unit, U.O Neurologia, IRCCS Ospedale S. Raffaele, Milano, Italy (G.G.).

**Keywords:** area under curve, atrial fibrillation, cerebral hemorrhage, coronary artery disease, myocardial infarction

## Abstract

**BACKGROUND:**

The identification of patients surviving an acute intracerebral hemorrhage who are at a long-term risk of arterial thrombosis is a poorly defined, crucial issue for clinicians.

**METHODS:**

In the setting of the MUCH-Italy (Multicenter Study on Cerebral Haemorrhage in Italy) prospective observational cohort, we enrolled and followed up consecutive 30-day intracerebral hemorrhage survivors to assess the long-term incidence of arterial thrombotic events, to assess the impact of clinical and radiological variables on the risk of these events, and to develop a tool for estimating such a risk at the individual level. Primary end point was a composite of ischemic stroke, myocardial infarction, or other arterial thrombotic events. A point-scoring system was generated by the β-coefficients of the variables independently associated with the long-term risk of arterial thrombosis, and the predictive MUCH score was calculated as the sum of the weighted scores.

**RESULTS:**

Overall, 1729 patients (median follow-up time, 43 months [25th to 75th percentile, 69.0]) qualified for inclusion. Arterial thrombotic events occurred in 169 (9.7%) patients. Male sex, diabetes, hypercholesterolemia, atrial fibrillation, and personal history of coronary artery disease were associated with increased long-term risk of arterial thrombosis, whereas the use of statins and antithrombotic medications after the acute intracerebral hemorrhage was associated with a reduced risk. The area under the receiver operating characteristic curve of the MUCH score predictive validity was 0.716 (95% CI, 0.56–0.81) for the 0- to 1-year score, 0.672 (95% CI, 0.58–0.73) for the 0- to 5-year score, and 0.744 (95% CI, 0.65–0.81) for the 0- to 10-year score. C statistic for the prediction of events that occur from 0 to 10 years was 0.69 (95% CI, 0.64–0.74).

**CONCLUSIONS:**

Intracerebral hemorrhage survivors are at high long-term risk of arterial thrombosis. The MUCH score may serve as a simple tool for risk estimation.

Although spontaneous intracerebral hemorrhage (ICH) accounts for only ≈10% of all strokes, it is the disease subtype with the most severe clinical consequences because of the associated high mortality and morbidity.^1^ In contrast to the much available data on patients’ outcome in the acute phase of the disease, the long-term prognosis of patients who survive after ICH has been poorly investigated to date. Incident and recurrent vascular events are a leading cause of death and long-term functional decline among ICH survivors.^2^ Emerging data suggest that patients with ICH may not only be at risk for recurrent cerebral bleeding but also at a higher risk of arterial ischemic events, particularly, ischemic stroke (IS) and ischemic cardiovascular disease, than the general population.^2^ Notwithstanding, mainly because of concerns about ICH recurrence, recommendations on the use of antithrombotic and statin medications for secondary prevention of arterial thrombotic events in these patients are still a matter of debate, even in selected ICH subgroups, such as, for example, those with specific comorbid conditions, like atrial fibrillation (AF).^3^ Stated another way, what, if any, secondary prevention therapy should be offered to ICH survivors is still a clinical dilemma. Because of this gap in knowledge, the identification of ICH survivors who are at the greatest risk of developing major vascular events during follow-up emerges as a crucial issue for clinicians.

MUCH-Italy (Multicenter Study on Cerebral Haemorrhage in Italy) provides the opportunity to investigate this clinical question owing to its large sample size, the homogeneous demographic characteristics and clinical phenotype of the subjects included, the standard diagnostic workup and follow-up evaluation. Therefore, in the present study, we aimed at (1) determining the long-term incidence of major arterial thrombotic events and bleeding recurrences in ICH survivors, (2) assessing the impact of clinical and radiological variables on the risk of these events, and (3) developing a multivariable risk score for estimating the long-term risk of arterial thrombotic events at the individual level, in a cohort of Italian patients with ICH.

## METHODS

The data supporting the findings of this study are available from the corresponding author upon reasonable request.

### Standard Protocol Approvals and Participant Consents

The institutional ethical standards committee on human experimentation at Brescia University Hospital provided approval for the study. Written informed consent was obtained for all participants (or next of kin).

### Study Design and Participant Selection

MUCH-Italy is a countrywide network of neurological centers designed to investigate the epidemiology, risk factors, and consequences of ICH in the setting of a hospital-based, multicenter, prospectively recruiting, observational cohort study.^4^ For the present analysis, we screened data sets from patients with acute ICH consecutively admitted from January 1, 2002, to July 31, 2014. Criteria for patient selection, risk factor definition, diagnostic procedures, and assessment of hematoma location have been described previously^4^ and are summarized in the Supplemental Methods. All patients underwent the first computed tomography scan at admission and a follow-up computed tomography scan at 24 hours of symptom onset. Hematoma expansion (HE) was defined as absolute growth of >6 mL or relative growth of >33% from the first computed tomography to the follow-up computed tomography.^5^

### Outcomes

Death was considered due to the index ICH if it occurred within 30 days of the onset of symptoms. Follow-up evaluations were conducted at 3 months and then annually, and outcome events were classified by using information from interviews (directly during follow-up visits or by telephone) with patients, next of kin, witnesses, and attending physicians or from hospital/general practitioner records. Subjects were included in the subgroup of patients who did not experience long-term vascular events if they had at least a 1-year follow-up.

The primary end point was a composite of fatal/nonfatal IS, MI, or other arterial thrombotic events. Secondary end points were (1) fatal/nonfatal IS, (2) fatal/nonfatal MI, (3) a composite of fatal/nonfatal recurrent ICH or major hemorrhagic events, and (4) recurrent fatal/nonfatal ICH.

Criteria for outcome definition are reported in the Supplemental Methods. If >1 event occurred during the follow-up, the first was used for calculation of the disease-free survival time. Long-term antithrombotic therapy and other treatment for secondary prevention were administered in accordance with published guidelines^6^ or, in cases where there was no clear evidence in favor of a treatment, at the discretion of the physician in charge of the patient. Adherence to secondary prevention medication (oral anticoagulants, aspirin or other antiplatelet agents, antihypertensive agents, oral hypoglycemic agents or insulin, and statins) and variations in vascular risk factors during follow-up were ascertained at each follow-up evaluation in the same way as for recurrent vascular events.

### Statistical Analysis

Duration of follow-up was calculated in person-months by using the follow-up of each participant from baseline examination until death, outcome event, or most recent censored follow-up assessment (corresponding to the end of the study, March 2022). We imputed the missing values (n=50) with mode (for binary variables) or mean (for continuous variables). Survival analyses were performed in the subgroup of patients who had survived at least 30 days after spontaneous ICH. The Kaplan-Meier survival analysis was used to estimate the cumulative incidence of outcome events by follow-up time.^7^ Hazard ratios (HRs) and 95% CIs were assessed by Cox proportional hazards models in univariable analyses to compare demographic variables and risk factor prevalence at baseline and in multivariable analysis, as well, to detect the independent predictors of outcome. We assessed the proportional hazards assumption for a Cox regression model fit by plotting estimates of the time-independent coefficient β versus time. If the proportional hazards assumption is true, each β is a horizontal line.^8^ The variables included in the regression model as covariates were selected a priori (ie, before model development) based on their assumed biological relevance according to the data of the literature. In each model, predictors of the overall primary and secondary end points were identified. The model included the following covariates: demographic characteristics (age and sex), traditional cardiovascular risk factors (hypertension, diabetes, hypercholesterolemia, active smoking, and alcohol consumption), comorbidities (personal history of ischemic heart disease and cancer, AF), characteristics of the index hemorrhage (hematoma location [deep versus lobar], HE), and use of secondary preventive medications (antithrombotic agents, statins). Because of the potential impact of hematoma location (deep versus lobar) and comorbid AF on the risk of vascular events after ICH and their possible therapeutical implications, we performed the same analysis stratifying the study group by site of hemorrhage and coexistent AF.

Based on the variables identified as predictors, we developed a risk score for the estimation of the individual long-term risk of arterial thrombotic events after ICH, using the least absolute shrinkage and selection operator variable selection procedure as proposed by Tibshirani^9^ in survival analysis. This is a penalized variable selection technique, which shrinks β-coefficients (β=ln[HR]) and produces some β-coefficients that are exactly zero. The variables whose β-coefficient is zero are then automatically deleted from the predictor set. Model screening was performed by tuning penalized parameter by K-fold cross-validation,^10^ with K=10 roughly equal-sized subsets. The nonzero β-coefficients of each predictor variable from the multivariable survival model with minimum least absolute shrinkage and selection operator penalty were used to generate a weighted scoring system of the predictors. An overall continuous individual risk score (MUCH score, s) for each patient (1) was calculated by summing up its β-coefficients×predictor values (x_j_) [s(i)=Ʃ_j_ β_j_x(i)_j_]. Exponential function, η(i)=exp[s(i)], represents the hazard score for each subject. Higher values of η(i) correspond to a higher level of hazard and a shorter survival time based on the predictors. To assess the predictive validity of the MUCH score, we used the receiver operating characteristic curves, the area under the receiver operating characteristic curve (AUC), and the discrimination C statistic (overall AUC), which take into account the timing of events from survival data^11–13^ (at 1, 5, and 10 years for AUC and from 0 to 10 years for C statistic). AUC and C summaries are 0-to-1 values, where 50% is the null value of worse scenario for decision-making. To account for the fact that we evaluated the risk score function on the same data on which it was developed, the C statistic (overall AUC) in predicting events that occur in a time range 0 to t was validated by K-fold cross-validation^10^ with K=10, each fold evaluating a test sample (n=173) by using scores obtained from the β-coefficients trained by the other learning sample (n=1729−173=1556). In this way, we corrected for potential overfitting in the assessment of the score performance. Additionally, we determined the MUCH score as predictor of arterial thrombotic events in the follow-up using Kaplan-Meier plots with significance testing by the log-rank test, after converting the score to a binary variable based on an optimal cutoff according to the Youden index method.^14^ Two-sided values of *P*<0.05 were considered significant. Statistical analyses were conducted with the R software (version 4.2.2, R Development Core Team: https://cran.r-project.org/).

## RESULTS

**Figure 1.**
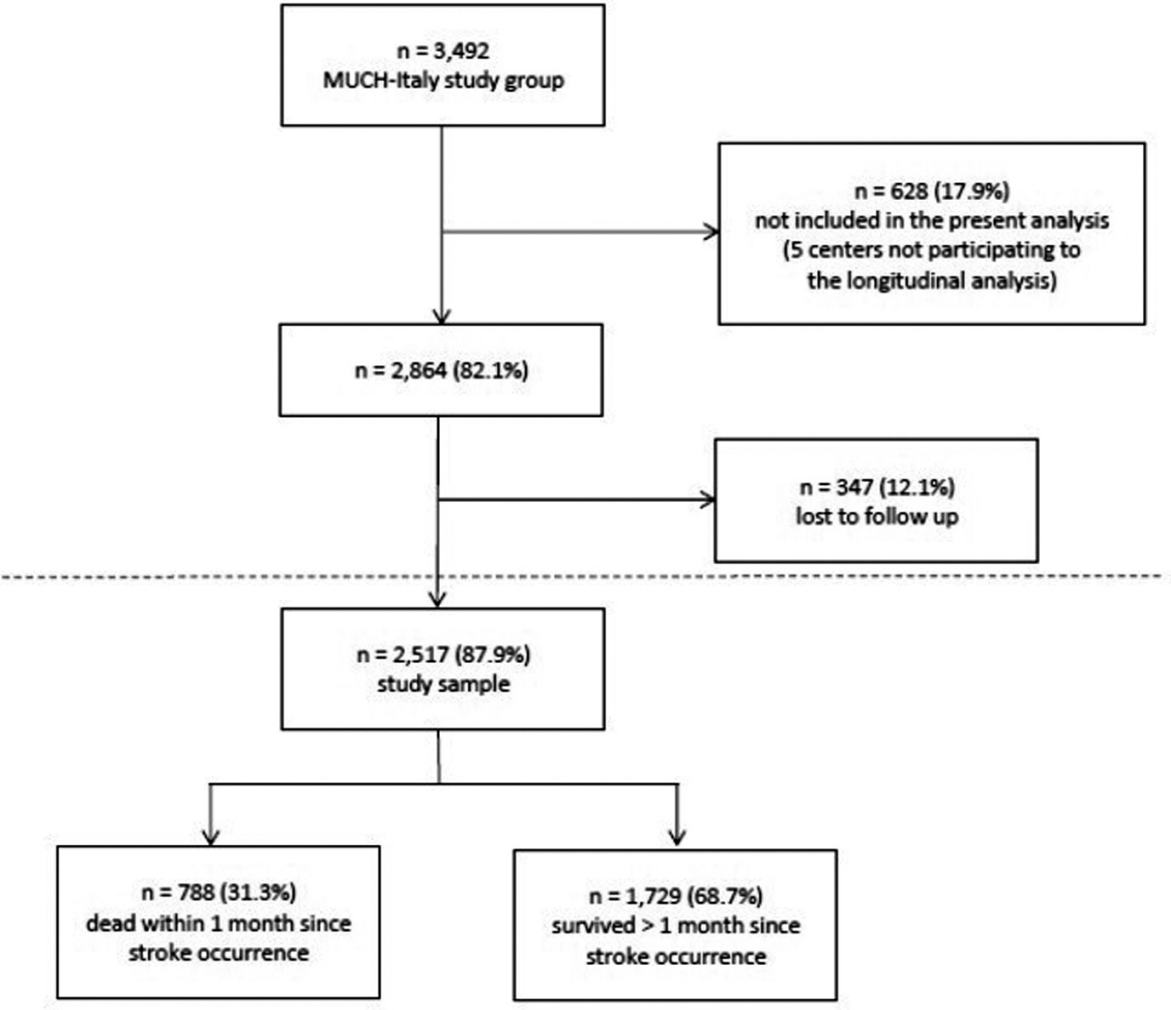
**Participants’ enrollment and eligibility criteria flowchart.** MUCH-Italy indicates Multicenter Study on Cerebral Haemorrhage in Italy.

Thirteen of the 19 centers comprising the MUCH-Italy network participated to the present study. The remaining 6 centers were unable to provide a systematic follow-up evaluation of the patients enrolled because of bureaucratic and administrative issues and, therefore, did not contribute to the present analysis. In all, of the 3492 patients with first-ever ICH included in the MUCH-Italy database, 2864 (82.0%) were considered for participation. Among them, 347 (12.1%) were excluded because they were lost to follow-up at the first postdischarge (3-month) evaluation, leaving 2517 (72.1%) patients (mean age, 73.8±12.5 years; males, 55.4%) eligible for inclusion in the present study (Figure 1). There were no significant differences in baseline demographic and clinical characteristics between the 2 subgroups, except for a higher prevalence of coronary artery disease among patients whose follow-up data were available for the analysis (not shown). Based on the location of the hematoma, 1170 (46.5%) patients were categorized as lobar ICH cases, while 1341 (53.3%) were considered deep (nonlobar) ICH cases (in the remaining 6 [0.2%] patients, a consensus on the exact hematoma location was not reached, and they were categorized as mixed ICH). HE was detected in 644 (26.6%) patients. Demographic and baseline clinical characteristics of the study group are summarized in Table 1.

**Table 1.**
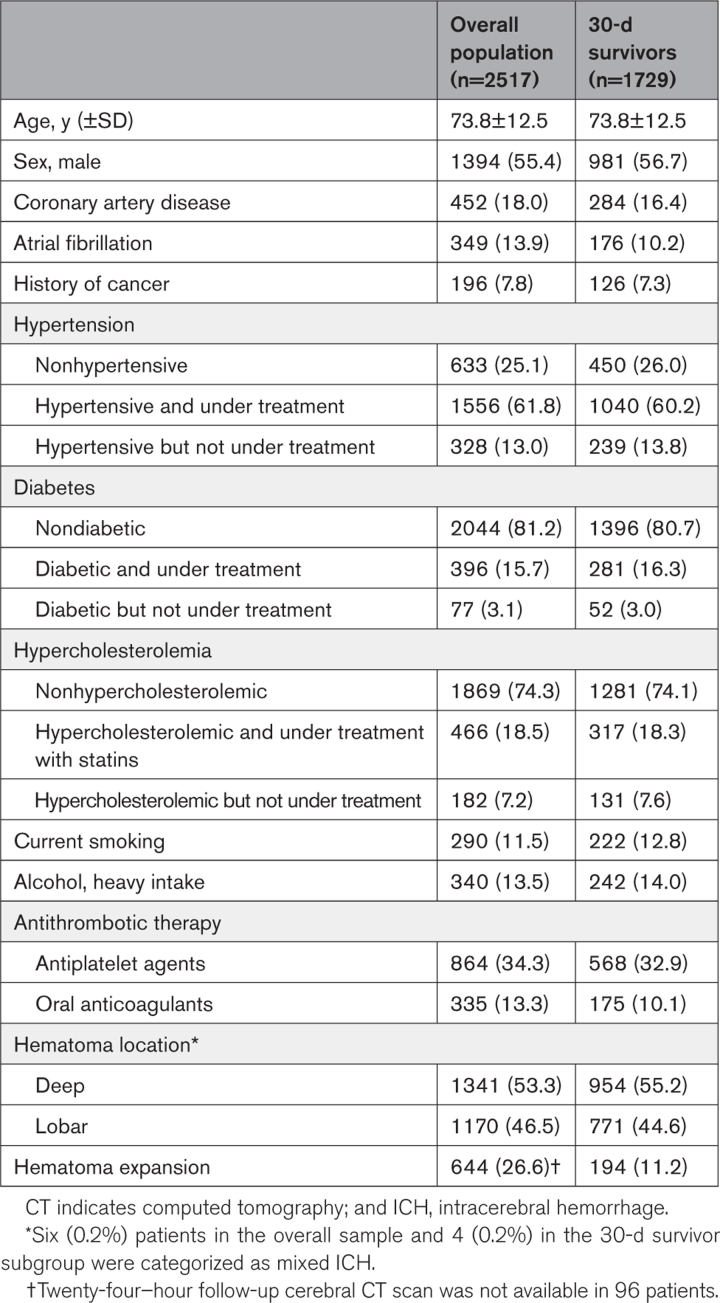
Demographic and Clinical Characteristics of the Study Group and the 30-Day Survivor Subgroup

Within the first 30 days after the index ICH, 788 (31.3%) patients died, leaving a group of 1729 (68.7%) participants available for long-term longitudinal analysis (Figure 1).

The latter subgroup was followed up for 7264 patient-years. The median follow-up time was 43 months (25th to 75th percentile, 69.0), with a maximum follow-up of 17 years. Changes in risk factor distribution and therapy at follow-up compared with baseline are reported in the Supplemental Methods and Figure S1.

### Outcome

Among the 1729 30-day survivors, we recorded major arterial thrombotic events in 169 (9.7%; 23 per 1000 patient-years) patients (108 [6.2%; 15 per 1000 patient-years] ISs, 39 [2.2%; 5 per 1000 patient-years] MIs, and 22 [1.2%; 3 per 1000 patient-years] other arterial thrombotic events) and major hemorrhagic events in 231 (13.3%; 32 per 1000 patient-years) patients (219 [12.7%; 30 per 1000 patient-years] recurrent ICH and 12 [0.6%; 2 per 1000 patient-years] other major hemorrhagic events) during the follow-up. Outcome events were confirmed by hospital/general practitioner records in 97.8% of the cases.

### Variable Selection and Risk Model Prediction

All models met Cox proportional hazards assumption. In multivariable Cox proportional regression analysis, 7 covariates predicted independently the risk of major arterial thrombotic events during the follow-up: male sex (HR, 1.49 [95% CI, 1.06–2.04]), diabetes (HR, 1.50 [95% CI, 1.07–2.10]), hypercholesterolemia (HR, 1.81 [95% CI, 1.30–2.54]), AF (HR, 2.29 [95% CI, 1.59–3.30]) and personal history of coronary artery disease (HR,1.91 [95% CI, 1.34–2.73]) turned out to increase this risk, whereas the use of statins (HR, 0.26 [95% CI, 0.15–0.46]) and antithrombotic medications (HR, 0.62 [95% CI, 0.38–0.99]; in particular, oral anticoagulants [HR, 0.22 (95% CI, 0.55–0.94)]) in the follow-up was associated with a reduced risk. Conversely, 3 covariates predicted independently the risk of major hemorrhagic events: increasing age (HR, 1.02 [95% CI, 1.01–1.03], for every 1-year increase of age), lobar location of the hematoma (HR, 1.72 [95% CI, 1.31–2.22]), and HE (HR, 2.17 [95% CI, 1.55–3.03]; Table 2).

**Table 2.**
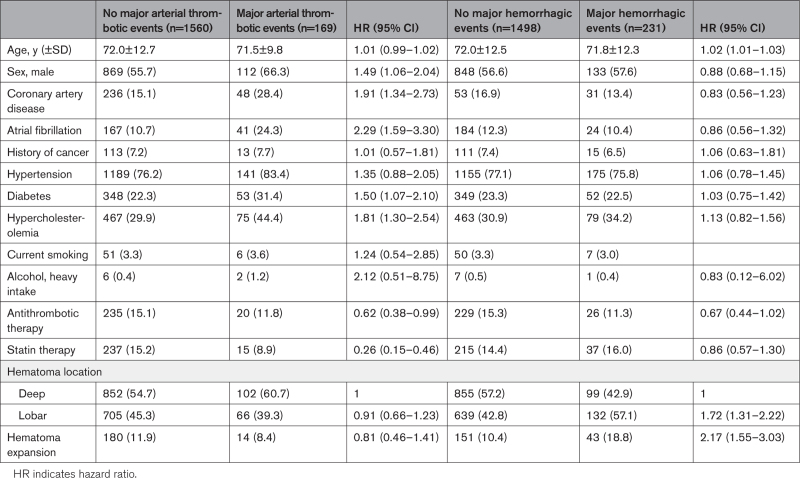
Multivariable Cox Proportional Hazards Model for Predicting the Primary End Points of Any Arterial Thrombotic Events and Any Major Hemorrhagic Events

The models with secondary end points (fatal/nonfatal IS, fatal/nonfatal ICH, and fatal/nonfatal MI) as outcome measure gave similar results (Table S1).

The least absolute shrinkage and selection operator technique for variable selection confirmed the nonzero β-coefficients of sex, antithrombotic medications use, diabetes, hypercholesterolemia, personal history of ischemic heart disease, AF, and statin use, as independent predictors of arterial thrombotic events during the follow-up. The MUCH score was generated by using the 7 predictor variables reported above. To derive a value for each parameter of the MUCH score, β-coefficients were rounded to the closest decimal (Table 3; the observed distribution of MUCH scores is summarized in Figure S2).

**Table 3.**
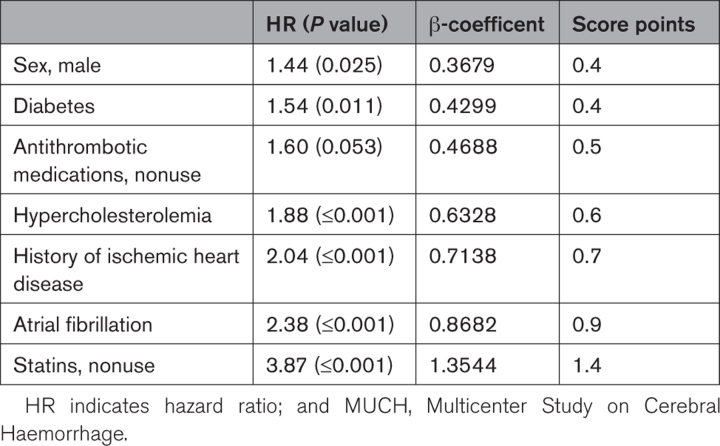
Multivariable Cox Proportional Hazards Model With the Predictors Selected by the Least Absolute Shrinkage and Selection Operator Procedure and the β-Weights (Last Column) of the MUCH Score Used for Computing the Individual Probability of Arterial Thrombotic Events After Intracerebral Hemorrhage

The sum of the weighted scores was used to estimate the overall score. This gave a continuous score whose values range between 0 and 5. The 1-, 5-, and 10-year individual risk is calculated using the following formulas: 1-year risk=1−0.997^exp(MUCH score)^; 5-year risk=1−0.93^exp(MUCH score)^, 10-year risk=1−0.978^exp(MUCH score)^.

### Assessment of Model Performance

**Figure 2.**
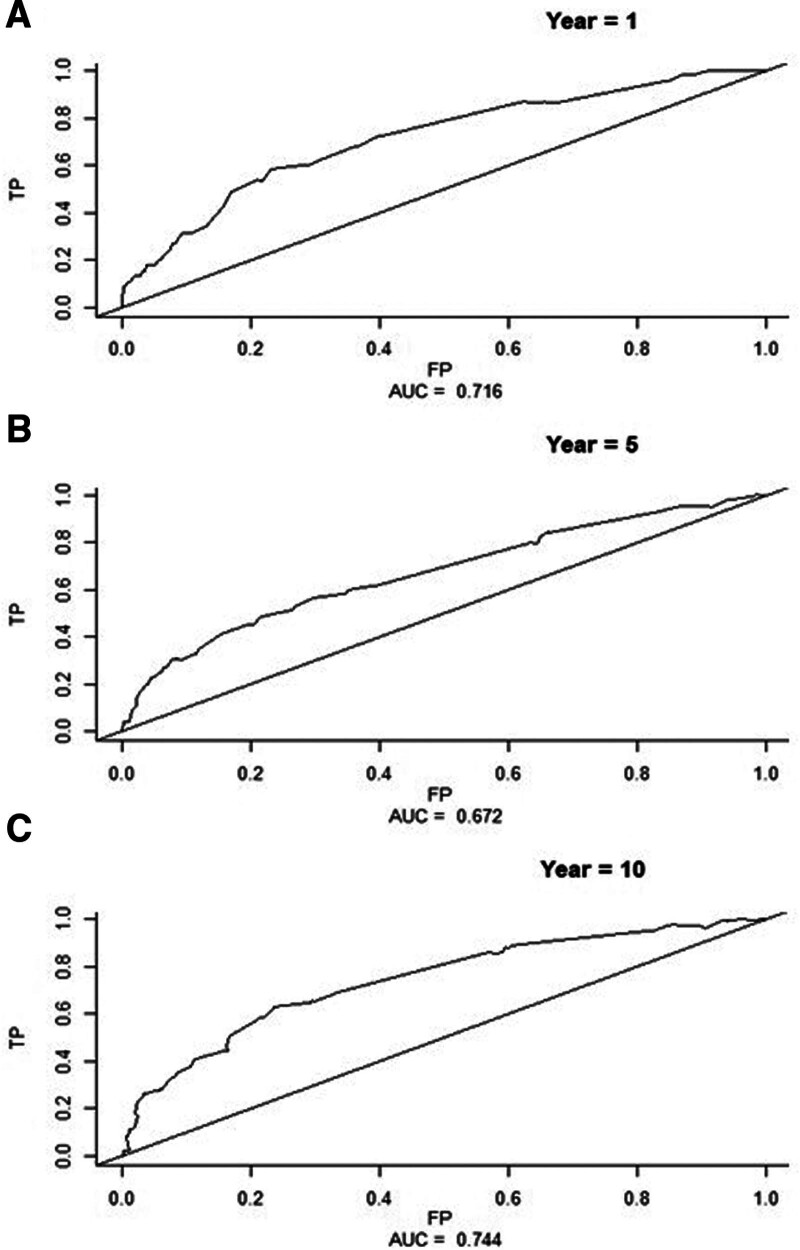
**Predictive performance of the MUCH (Multicenter Study on Cerebral Haemorrhage) score.** Receiver operating characteristic (ROC) curves to obtain the C statistic for arterial thrombotic events at 1 y (**A**), 5 y (**B**), and 10 y (**C**). AUC indicates area under the receiver operating characteristic curve; FP, false positive; and TP, true positive.

The MUCH score offered moderate discrimination for the long-term risk of arterial thrombotic events. In particular, the AUCs were 0.716 (95% CI, 0.56–0.81) at 1 year, 0.672 (95% CI, 0.58–0.73) at 5 years, and 0.744 (95% CI, 0.65–0.81) at 10 years (Figure 2), indicating acceptable predictive performance of our model. Discrimination C statistic for the prediction of events that occur in the time range of 0 to 10 years was 0.691 (95% CI, 0.64–0.74).

**Figure 3.**
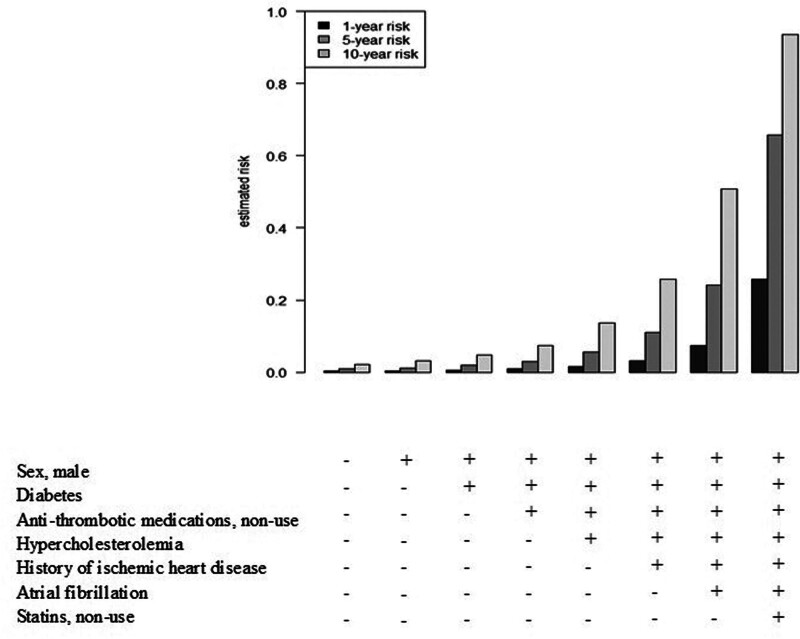
**One-y vs 5-y vs 10-y risk of arterial thrombotic events after intracerebral hemorrhage for subjects with different risk profiles.** 1-year risk, 1−0.997 ^exp(MUCH score)^; 5-year risk, 1−0.992 ^exp(MUCH score)^; 10-year risk, 1−0.978 ^exp(MUCH score)^.

Mean 10-fold cross-validated C statistic was 0.64, suggesting that the bias coming from predicting on the same data set used for fitting was ≈0.1. Kaplan-Meier plots comparing the 2 groups of patients defined by the optimal cutoff value of the MUCH score (<2.62 versus >2.62) confirmed the predictive performance of the score (log-rank test; χ^2^[degree of freedom]=70.8 [1]; *P*<0.001; Figure S3). Figure 3 contrasts the estimated 1-, 5-, and 10-year risk of arterial thrombotic events in the follow-up in patients with varied combinations of predictors. For each combination, the 5-year model gives risk estimates that are 2 to 3× higher and the 10-year model ≈4× higher than those of the 1-year model. For example, the 1-year risk for a patient with all the 7 predictors is ≈25%, but the corresponding 5-year risk reaches ≈65% and the 10-year risk reaches ≈95%.

The rate of major arterial thrombotic events did not differ by ICH location, but it was higher among patients with comorbid AF than among those without (Supplemental Results).

## DISCUSSION

Approximately 2.9 million people worldwide every year are affected by ICH, with obvious socioeconomic consequences in terms of life-years with disability and life-years lost.^1^ The burden of disease rises even more in the case of recurrent vascular events, which emphasizes the need for appropriate secondary prevention approaches. The results of our observational study, including a large cohort of well-characterized Italian patients, indicate that subjects who survive the first 30 days after an ICH are at substantial risk of major vascular events over time. In particular, the risk of arterial thrombosis is partly attributable to modifiable factors, among which the lack of resumption or initiation of antithrombotic and statin medications, if indicated for secondary prevention of major vascular events, has a prominent role and can be estimated in a clinical setting by a simple prediction algorithm based on the combination of these factors. Comorbid AF was the principal risk factor for arterial thrombosis and IS but had no influence on the risk of major hemorrhagic events after the index ICH, while lobar ICH location and HE were the principal risk factors for recurrent ICH but did not impact the subsequent risk of arterial thrombosis. Conversely, although high blood pressure is an established and strong risk factor for vascular disease, we failed to show an association between hypertension and long-term risk of vascular events after ICH. The interpretation of this finding is far from obvious. There is a possibility that initiation of blood pressure treatment in patients with ICH may attenuate a possible association between premorbid hypertension and risk of vascular events in the long term. Studies on secondary prevention, for example, have shown an association with lowering of blood pressure and reduced recurrence rates of ICH.^6^

Another notable finding of the present analysis is that resumption or initiation of antithrombotic medications after ICH reduces the long-term risk of ischemic or thromboembolic events, especially IS, without increasing that of major rebleedings. Statins would appear to have the same effect. The benefits of disrupting the formation of platelet plugs and coagulation cascade with antithrombotic agents and intensive lowering of cholesterol with statins are well established in patients with IS and coronary artery disease. However, antithrombotic drugs also increase the risk of bleeding, as a result of their effect on clotting and platelet aggregation. In this regard, our findings are in line with those of the only completed randomized controlled trial of antiplatelet therapy after ICH^15,16^ and move in the same direction of those of the largest randomized trial of oral anticoagulation for AF after ICH^17^ published to date. Similarly, the best strategy for treating dyslipidemia after ICH is currently unknown, given the potential of increased propensity for recurrent ICH with statin therapy. Therefore, whether or not to use these medications in ICH survivors is a common therapeutic dilemma in everyday clinical practice. Because of the few randomized controlled data currently available,^18,19^ our results, derived from a large prospective data set, might have implications for clinicians, since they provide reassurance to continue or initiate antithrombotic medications and statins after ICH, without significant harm.

Most of the studies conducted so far on the long-term risk of arterial thrombosis after ICH do not allow reliable estimates for a number of reasons. Some of them were clearly underpowered for multivariable analysis because of the rather modest number of patients involved and the short length of follow-up^2^; others lacked data on antithrombotic therapy or location of the cerebral hematoma,^20^ captured end points that were a composite of thrombotic and hemorrhagic events making it hard to derive precise information on arterial thrombosis; and a few had limited generalizability because of the highly selected cohorts under investigation.^2,20,21^ Recently, a pooled analysis of 4 US population-based studies with ≈50 000 participants provided novel findings implicating ICH as a potential risk marker for subsequent arterial ischemic events.^22^ Notwithstanding, as admitted by the authors themselves, the study could not explore the association between hematoma location and the risk of subsequent arterial thrombosis because of the lack of data or allow to perform subgroup analyses of recurrent ICH owing to the low number of such events. In addition, IS and MI were the only outcomes that were censored in that study, which, therefore, cannot provide information about the risk of other major arterial thrombotic events over time. Similarly, data derived from the Danish Stroke Registry confirmed that patients with a prior ICH had higher rates of major ischemic (except MI) and hemorrhagic vascular events, especially in the first year after an ICH compared with the general population, thus emphasizing the need for further research into effective secondary preventive measures to reduce these risks, but the study lacked data on ICH location and could not provide information on the underlying cause of ICH.^23^ Since many of the limitations mentioned above do not apply to our study, the present analysis yields essential new information on the long-term risk of arterial thrombosis after ICH. In particular, it provides rather stable results on the specific predictors of clinical events in the long term. Factors that turned out to predict thrombotic risk in our cohort of patients with ICH (male sex, diabetes, hypercholesterolemia, history of ischemic heart disease, AF, noncompliance with secondary preventive medications) are, actually, among those with the highest impact on thrombotic risk even in people with no history of ICH, just as the coexistence of multiple factors in one subject is well known to increase the long-term propensity to arterial thrombosis. These findings, therefore, might be regarded as not entirely unexpected. Notwithstanding, whether risk factors for thrombosis in the general population are also risk factors in ICH survivors has never been definitively proven. In addition, identifying predictors of arterial thrombotic events after ICH could help with risk stratification to inform decisions about secondary prevention strategies. In this regard, the score we had developed based on these factors can be a useful tool in a clinical setting because of its characteristics of simple prediction algorithm for the estimation of the individual long-term risk.

Effective risk communication is another reason why our long-term risk prediction score might be helpful. ICH survivors are more likely to adopt lifestyle changes on hearing that their long-term risk of arterial thrombosis is ≈30% than when they are told it is ≈3%.

In line with the results of several hospital-based studies and those of a recent population-based study,^2,24^ our findings also showed that hematoma location is a strong, independent predictor of recurrent ICH but has no effect on the long-term propensity to arterial thrombosis. This on the one hand emphasizes the potential bleeding-promoting effect of the underlying cerebral amyloid angiopathy in cases of lobar ICH location and on the other hand suggests that the magnitude of the prothrombotic effect after ICH does not differ by ICH location.

### Strengths and Limitations

MUCH-Italy combines the advantages of a prospective, multicentric, hospital-based recruitment, enabling a large sample size with detailed and standardized data collection with few missing data, with a long-term systematic assessment of all major vascular events. Notably, as opposed to most previous studies that recorded data on vascular risk factors and secondary prevention therapy at patient discharge, our study provides information on their eventual variations or occurrence, as well as their treatment during follow-up at the individual level. Moreover, it encompasses stroke recurrences but also extracerebral thrombotic and hemorrhagic events among the outcome measures and incorporates specific ICH characteristics (such as hematoma location and HE) in the prediction model. But our study also has some limitations. First, it covers a long period of time, which makes it subject to variability in vascular imaging technology, estimation of the prevalence of historical risk factors, and therapeutic options. Furthermore, data on some variables (ie, smoking habit and alcohol intake) were self-reported, which cannot exclude the unavoidable risk of misclassification. Second, we do not have reliable information on how effectively risk factors were controlled in the follow-up. For example, although long-term compliance with antihypertensive therapy was high in our cohort with ≈100% of patients regularly taking the recommended medication, it does not necessarily implicate adequate reduction of blood pressure in clinical practice. In addition, the study protocol did not include any formal assessment, other than the clinical interview with the patient, of individual noncompliance with antithrombotic medications or with international normalized ratio values at follow-up evaluation, which may have artificially increased the long-term risk of arterial thrombosis. Third, we cannot exclude that the long-term effect of resuming antithrombotic medications and statins we observed might be prone to confounding by indication, and, hence, these findings need to be confirmed by those of the randomized trials currently underway (https://www.clinicaltrials.gov; unique identifier: NCT03950076, NCT03243175, NCT03907046, and NCT03936361). Fourth, while the MUCH score has an acceptable predictive performance at 1 year and at 10 years, it appears less reliable at 5 years, which, in clinical practice, implies caution when using this tool for midterm prediction. Moreover, although we have internally validated the MUCH score by 10-fold cross-validation, external validation on an independent cohort would further strengthen its clinical value. Fifth, since we did not include the magnetic resonance imaging data in our analysis, we cannot estimate how relevant imaging biomarkers of cerebral small vessel disease at baseline might be in predicting the risk of vascular events over time. Finally, our results are based on an Italian population and might not be generalizable to other countries or ethnicities.

### Conclusions

In a large cohort of Italian patients with ICH, we found that the risk of subsequent arterial thrombotic events is associated with modifiable risk factors, the most prominent of which is comorbid AF, and it can be reliably estimated in clinical setting by a simple risk score based on the combination of these factors. Our findings, in particular, emphasize the importance of extending the use of secondary prevention treatments (ie, antithrombotic therapy and statin medications) into the long term. Implementation of appropriate therapeutic and lifestyle treatment strategies in these patients is likely to impact the individual susceptibility to vascular recurrence.

## ARTICLE INFORMATION

### Sources of Funding

None.

### Disclosures

Dr Ciccone reports grants from Daiichi Sankyo, Italfarmaco, and Alexion Pharmaceuticals. Dr Paciaroni reports compensation from Sanofi-Aventis U.S. LLC, Pfizer Canada, Inc, iRhythm Technologies, Daiichi Sankyo Europe GmbH, and Bristol Myers Squibb for other services. The other authors report no conflicts.

### Supplemental Material

Supplemental Methods

Supplemental Results

Figures S1–S3

Table S1

References^25–28^

## Supplementary Material
