# Supplementary material for: Long-Term Risk of Arterial Thrombosis After Intracerebral Hemorrhage: MUCH-Italy
Source: Stroke. 2024 Feb 1;55(3):634–42. doi: 10.1161/STROKEAHA.123.044626 (PMC10896192; doi:10.1161/STROKEAHA.123.044626)
Supplement: Supplementary file 1 [file str-55-634-s001.pdf]

Supplemental Material

Long-term risk of arterial thrombosis after intracerebral haemorrhage: ~~The~~ MUCH-Italy

Alessandro Pezzini et al

Supplemental Methods

Supplemental Results

Figure S1

Changes of risk factor distribution and therapy in the follow-up

Figure S2

Observed distribution of MUCH scores in the study cohort.

Figure S3

Kaplan-Meier estimates of arterial thrombotic events in the group of patients with the lowest vs highest MUCH-score defined by the optimal Youden cut-off on the 1-year ROC.

Testing of significance is by the log-rank test.

Table S1

Multivariable Cox proportional hazard model for predicting the secondary end-points ischemic stroke, myocardial infarction and recurrent intracerebral haemorrhage.

Supplemental Methods

Eligibility for study participation required neuroimaging (CT or MRI) confirmation of haemorrhagic stroke. Exclusion criteria included the presence of trauma, brain tumour, haemorrhagic transformation of a cerebral infarction, vascular malformation or any other perceived cause of secondary ICH. Hematoma location was assigned based on admission CT scan by stroke neurologists at each participating centre. ICH isolated to the cortex (with or without involvement of subcortical white matter) and cerebellar haematomas were defined as lobar ICH, while ICH selectively involving the thalamus, basal ganglia or brainstem was defined as deep (non-lobar) ICH. In case of multiple concurrent bleeds involving both deep and lobar territories (mixed ICH) ICH location was assessed by consensus.

Risk factor definition

A history of vascular risk factors was defined as the presence of these predisposing conditions, either in the personal medical history or when identified during patients' hospital stay. Smoking was defined as currently smoking one or more cigarettes per day on a regular basis. Hypertension was defined as systolic blood pressure, BP >140 mm Hg and/or diastolic BP >90 mm Hg of the acute phase or using pharmacological treatment for hypertension. Diabetes was defined as fasting glucose levels >6.9 mmol/L of the acute phase or current treatment with antidiabetic drugs. Based on daily alcohol consumption, participants were dichotomised into excessive drinkers (>45 g of alcohol) and light-moderate drinkers or non-drinkers. We also collected information on AF (medical history or electrocardiographic findings at admission), atherosclerotic peripheral arterial disease (medical history), coronary artery disease (medical history of angina, MI, coronary artery bypass graft or percutaneous transluminal coronary angioplasty), and pre-ICH medications (warfarin or direct oral anti-coagulants [DOACs], aspirin or other antiplatelet agents, antihypertensive agents, oral hypoglycaemic agents or insulin and statins).

Changes of risk factor distribution and therapy in the follow-up

Fifty-one (2.9%) patients who were non-hypertensive at the time of the index ICH got the diagnosis of hypertension in the follow-up, diabetes was diagnosed in 68 (3.9%) previously non-diabetic patients, hypercholesterolemia in 94 (5.4%) previously non-hypercholesterolemic patients, and a new diagnosis of AF was made in 32 (1.8%) patients. Of the 222 patients who were active smokers before ICH occurrence, 167 (75.2%) quit smoking, 55 (24.8%) kept smoking, while 2 patients who were non-smokers at the time of stroke started smoking afterwards, thus, yielding a total of 57 (3.3%) active smokers during the follow-up. Two-hundred-thirty-four (99.6%) patients with a personal history of heavy alcohol consumption before the index stroke significantly reduced or discontinued alcohol intake, leaving a small subgroup of 8 (0.4%) patients who went on to consume pathological amounts of alcohol after ICH occurrence. All hypertensive patients and all diabetic patients received specific therapy during follow-up. One-hundred-seventy-one (53.9%) patients taking statins at the time of stroke onset did not resume these medications after the index ICH, while 106 patients who were not receiving statins before the index stroke started taking them later on, thus leading to 252 (14.5%) patients being treated with statins in the follow-up. The median time to resumption/initiation of statins after the incident ICH was 1.0 months (25th to 75th percentile, 12.0). Finally, among the 568 patients who were under treatment with antiplatelet agents at the time of the index stroke 419 (73.8%) stopped taking any anti-thrombotic drugs during follow-up, 136 (24.0%) resumed anti-platelets, 6 (1.0%) switched to warfarin and 7 (1.2%) to DOACs. Among the 175 patients previously taking warfarin, 106 (60.6%) discontinued any anti-thrombotic medications, 55 (31.4%) switched to anti-platelets, 6 (3.4%) to DOACs, and 8 (4.6%) resumed warfarin. Thirty-seven patients who were not using any anti-thrombotic drug at the time of the index ICH started taking these agents thereafter (n = 28 antiplatelets; n = 5 warfarin; n = 4 DOACs). Overall, therefore, 219 (12.6%) patients were on anti-platelet therapy and 36 (2.1%) on oral anti-coagulant therapy in the follow-up (Supplemental Figure I). The median time to

resumption/initiation of anti-thrombotic medications after the incident ICH was 3.0 months (25th to 75th percentile, 11.0).

Outcome definition

IS was defined as a sudden loss of global or focal cerebral function that persisted for >24 hours with a probable vascular cause[25] with brain imaging (CT or MRI) confirmation of the infarcted area. IS due to sinus venous thrombosis, vasospasm after subarachnoid haemorrhage, cardiac surgery, occurring as an immediate consequence of trauma, and iatrogenic strokes were excluded. MI was diagnosed when at least 2 criteria among (1) chest pain, (2) characteristic ECG changes, and (3) cardiac enzyme abnormalities[26] were present. Arterial thrombotic events other than IS and MI comprised the following: acute coronary syndrome other than MI, acute limb ischemia, coronary artery revascularization (either coronary artery bypass or percutaneous coronary intervention), severe peripheral artery disease (ie, symptomatic arterial stenosis or stenosis requiring a revascularization procedure), and severe internal carotid stenosis requiring a revascularization procedure[27]. Conversely, we did not include covert vascular brain lesions (either ischemic or haemorrhagic) and TIAs.

Recurrent ICH was defined by using the same criteria applied for the definition of the index event. Major haemorrhagic events other than spontaneous ICH were classified using the Bleeding Academic Research Consortium. Major bleedings were defined as type ≥3 (type 3a: bleeding with haemoglobin drop of 3–5 g/dL or needing blood transfusion; type 3b: bleeding with haemoglobin drop of ≥5 g/dL or requiring surgical intervention for control or intravenous vasoactive agents; type 3c: imaging confirmed intracranial [ie, subdural, extradural, subarachnoid, pure intraventricular haemorrhages, post-traumatic ICH], intraspinal haemorrhage or intraocular bleed; type 4: coronary bypass-related bleeding; type 5: fatal bleeding)[28].

Supplemental Results

The median time to major haemorrhagic events after the incident ICH (30 months; 25th to 75th percentile, 48.0) was shorter than that to arterial thrombotic events (45.0 months; 25th to 75th percentile, 71.0) and the latter was shorter for IS (36 months; 25th to 75th percentile, 62.3) than for MI (54 months; 25th to 75th percentile, 63.0).

Deep ICH vs lobar ICH

Patients with lobar ICH were older than patients with non-lobar ICH (mean age, 72.7±12.6 years vs 71.4±12.4; p=0.039), less frequently males (417 [54.1%] *vs* 561 [58.8%]; p=0.049) and more likely to have been diagnosed with ischemic heart disease (143 [18.5%] *vs* 141 [14.8%]; p=0.036), but there were no other baseline differences by ICH location. The rate of recurrent haemorrhagic events was higher after lobar ICH (132 [17.1%]) than after non-lobar ICH (99 [10.4%]; p≤0.001; HR, 1.72; 95% CI, 1.31-2.22, in multivariable Cox proportional regression analysis), and so was the rate of recurrent ICH (128 [16.6%] *vs* 91 [9.5%]; p≤0.001; HR, 1.77; 95% CI, 1.35-2.33). However, the rate of major arterial thrombotic events (66 [8.6%] after lobar ICH vs 102 [10.7%] after non-lobar ICH; p=0.138; HR, 0.91; 95% CI, 0.66-1.23), IS (42 [5.4%] after lobar ICH vs 65 [6.8%] after non-lobar ICH; p=0.242; HR, 0.88; 95% CI, 0.59-1.31) and MI (13 [1.7%] after lobar ICH vs 26 [2.7%] after non-lobar ICH; p=0.192; HR, 0.76; 95% CI, 10.38-1.51) did not differ by ICH location.

Comorbid AF vs no AF

Patients with comorbid AF were older (mean age, 76.4±9.9 years vs 71.4±12.7; p≤0.001), more likely to have a personal history of ischemic heart disease (58 [27.9%] vs 226 [14.9%]; p≤0.001), hypertension (175 [84.1%] vs 1155 [75.9%]; p=0.008), to take anti-thrombotic medications (43 [20.7%] vs 212 [13.9%]; p=0.010) and to be affected by HE (33 [16.6%] vs 161 [10.8%]; p=0.017) than those without AF. The rate of IS among patients with comorbid AF (32 [15.4%]) was higher

compared to that among patients without comorbid AF (76 [5.0%]; p≤0.001; HR, 2.96; 95% CI, 1.91-4.59, in multivariable Cox proportional regression analysis) and so was that of major arterial thrombotic events (41 [19.7%] vs 128 [8.4%]; p≤0.001; HR, 2.29; 95% CI, 1.59-3.30), but comorbid AF did not influence the rate of MI (8 [3.8%] vs 31 [2.0%]; p=0.128; HR, 1.74; 95% CI, 0.76-3.94), major haemorrhagic events (24 [11.5%] vs 207 [13.6%]; p=0.410; HR, 0.86; 95% CI, 0.56-1.32) or recurrent ICH (19 [9.1%] vs 200 [13.1%], p=0.103; HR, 0.69; 95% CI, 0.43-1.12).

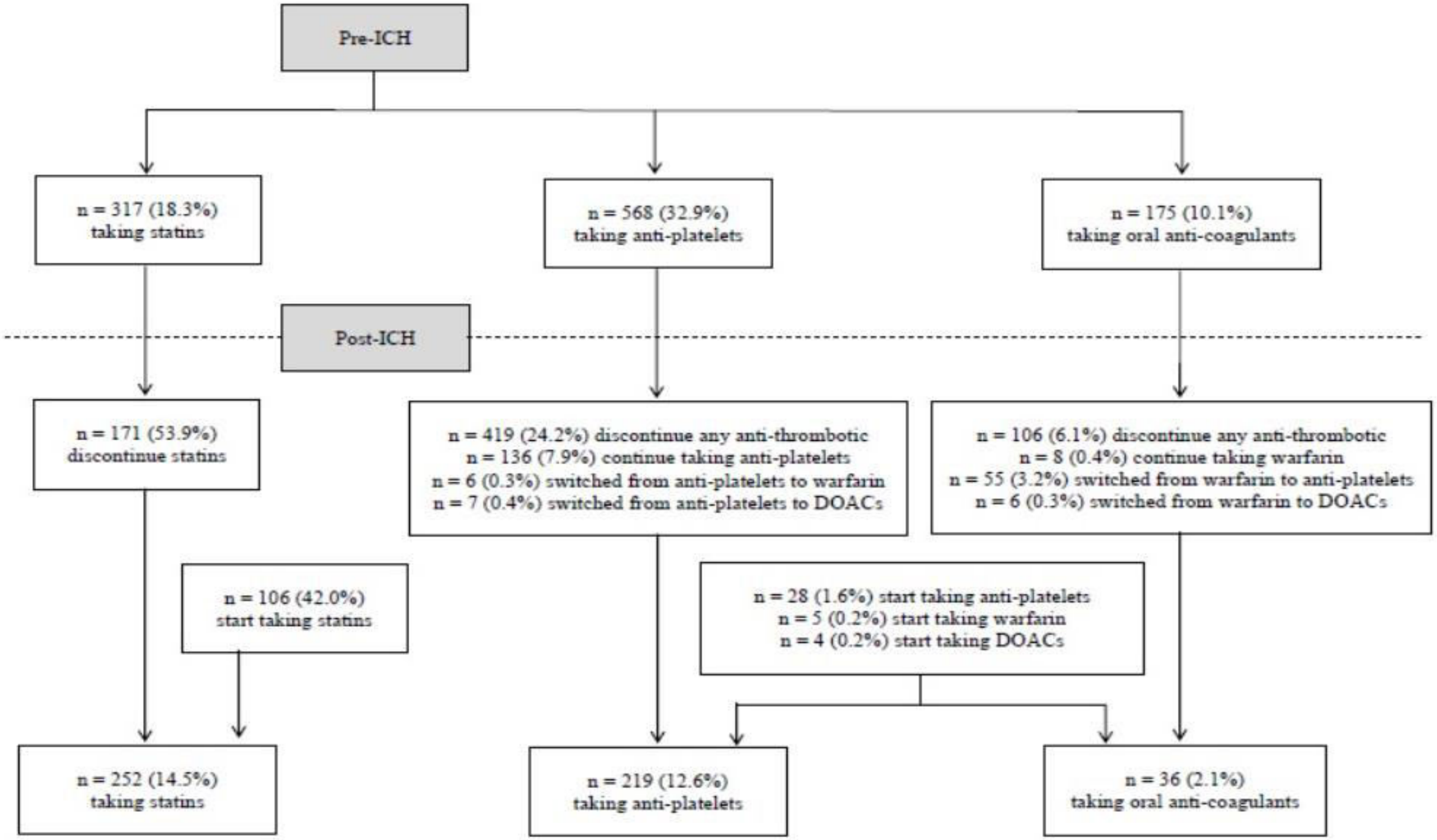
Pre-ICH
n = 317 (18.3%) taking statins
n = 568 (32.9%) taking anti-platelets
n = 175 (10.1%) taking oral anti-coagulants
Post-ICH
n = 171 (53.9%) discontinue statins
n = 419 (24.2%) discontinue any anti-thrombotic
n = 136 (7.9%) continue taking anti-platelets
n = 6 (0.3%) switched from anti-platelets to warfarin
n = 7 (0.4%) switched from anti-platelets to DOACs
n = 106 (6.1%) discontinue any anti-thrombotic
n = 8 (0.4%) continue taking warfarin
n = 55 (3.2%) switched from warfarin to anti-platelets
n = 6 (0.3%) switched from warfarin to DOACs
n = 106 (42.0%) start taking statins
n = 28 (1.6%) start taking anti-platelets
n = 5 (0.2%) start taking warfarin
n = 4 (0.2%) start taking DOACs
n = 252 (14.5%) taking statins
n = 219 (12.6%) taking anti-platelets
n = 36 (2.1%) taking oral anti-coagulants

Figure S1

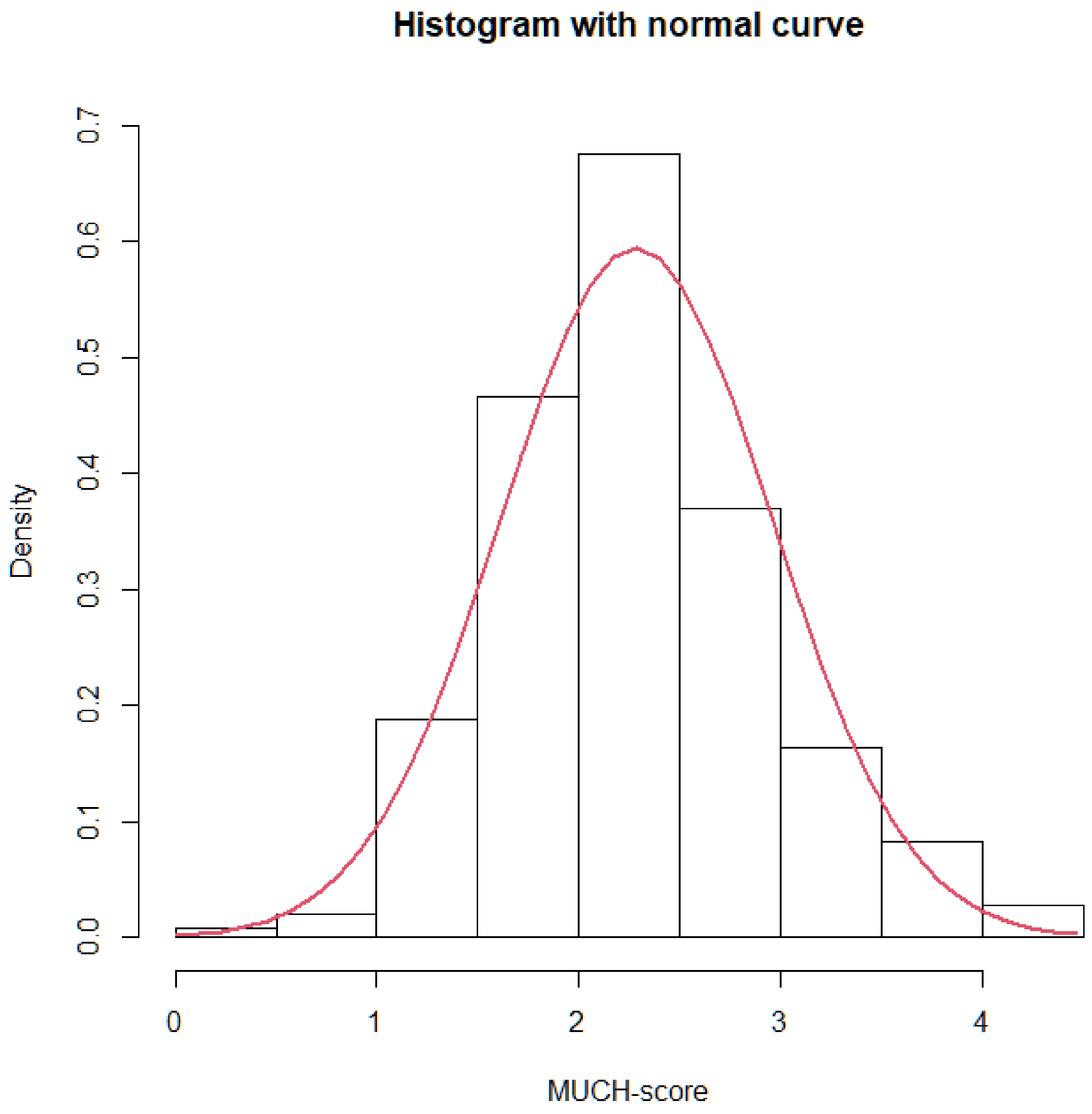
Histogram with normal curve
Density
0.0
0.1
0.2
0.3
0.4
0.5
0.6
0.7
0
1
2
3
4
MUCH-score

Figure S2

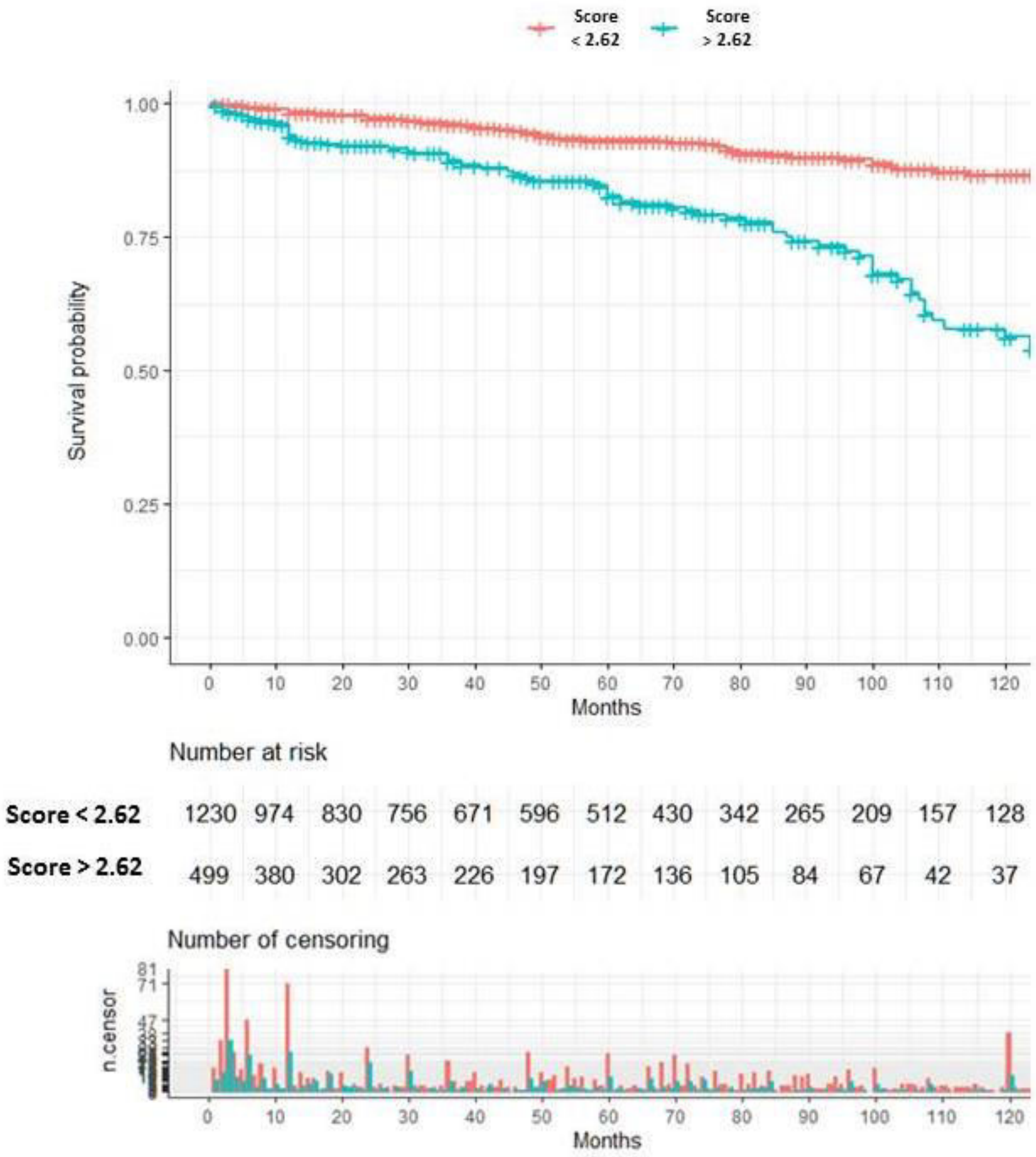
Score < 2.62
Score > 2.62
Survival probability
1.00
0.75
0.50
0.25
0.00
0 10 20 30 40 50 60 70 80 90 100 110 120
Months
Number at risk
Score < 2.62 1230 974 830 756 671 596 512 430 342 265 209 157 128
Score > 2.62 499 380 302 263 226 197 172 136 105 84 67 42 37
Number of censoring
n.censor
81
71
47
0 10 20 30 40 50 60 70 80 90 100 110 120
Months

Figure S3

	Ischemic stroke (n = 108)	HR (95% CI)	Myocardial infarction (n = 39)	HR (95% CI)	Recurrent ICH (n = 219)	HR (95% CI)
**Age, yrs ± SD**	72.1 ± 9.7	1.01 (0.99 - 1.03)	70.7 ± 9.3	0.99 (0.96 - 1.02)	71.6 ± 12.3	1.01 (1.003 - 1.02)
**Sex, Male**	63 (67.6)	1.63 (1.06 - 2.49)	28 (71.8)	0.57 (0.27 - 1.17)	125 (57.1)	0.92 (0.70 - 1.22)
**Coronary artery disease**	27 (25.0)	1.68 (1.05 - 2.69)	18 (46.2)	3.16 (1.57 - 6.38)	30 (13.7)	0.86 (0.57 - 1.29)
**Atrial fibrillation**	32 (29.6)	2.96 (1.91 - 4.59)	8 (20.5)	1.74 (0.76 - 3.94)	19 (8.7)	0.69 (0.43 - 1.12)
**Hystory of cancer**	8 (7.4)	0.95 (0.45 - 1.99)	3 (7.7)	0.89 (0.26 - 3.01)	15 (6.8)	1.13 (0.66 - 1.92)
**Hypertension**	88 (81.5)	0.82 (0.49 - 1.38)	34 (87.2)	0.74 (0.27 - 1.97)	167 (76.3)	0.94 (0.68 - 1.30)
**Diabetes**	38 (35.2)	0.52 (0.34 - 1.01)	10 (25.6)	1.05 (0.49 - 2.24)	50 (22.8)	0.95 (0.68 - 1.32)
**Hypercholesterolemia**	39 (36.1)	1.22 (0.78 - 1.91)	30 (76.9)	7.49 (3.42 - 16.42)	75 (34.2)	1.10 (0.79 - 1.54)
**Current smoking**	4 (3.7)	0.76 (0.27 - 2.09)	0 (0.0)	_	7 (3.2)	0.88 (0.41 - 1.88)
**Alcohol, heavy intake**	1 (0.9)	1.83 (0.24 - 13.48)	0 (0.0)	_	0 (0.0)	_
**Antithrombotic therapy**	8 (7.4)	0.38 (0.18 . 0.79)	5 (12.8)	0.55 (0.20 - 1.50)	24 (11.0)	0.69 (0.45 - 1.07)
**Statin therapy**	9 (8.3)	0.33 (0.16 - 0.70)	3 (7.7)	0.12 (0.03 - 0.41)	36 (16.4)	0.87 (0.56 - 1.33)
**Hematoma location***						
Deep	65 (60.7)	1.13 (0.76 - 1.69)	26 (66.7)	1.31 (0.66 - 2.59)	91 (41.6)	0.56 (0.42 - 0.74)
Lobar	42 (39.3)		13 (33.3)		128 (58.4)	1
**Hematoma expansion**	11 (10.4)	0.99 (0.52 - 1.86)	3 (7.9)	0.83 (0.25 - 2.79)	41 (18.9)	2.16 (1.53 - 3.05)

Table S1
